# Myc and mRNA capping^☆^

**DOI:** 10.1016/j.bbagrm.2014.03.007

**Published:** 2015-05

**Authors:** Sianadh Dunn, Victoria H. Cowling

**Affiliations:** MRC Protein Phosphorylation Unit, College of Life Sciences, University of Dundee, Dow Street, Dundee DD1 5EH, UK

**Keywords:** c-Myc, Capping, mRNA, 7-methylguanosine, Transcription

## Abstract

c-Myc is upregulated in response to growth factors and transmits the signal to proliferate by altering the gene expression landscape. When genetic alterations result in growth factor-independent c-Myc expression, it can become an oncogene. The majority of human tumour types exhibit a degree of c-Myc deregulation, resulting in unrestrained cell proliferation. c-Myc binds proximal to the promoter region of genes and recruits co-factors including histone acetyltransferases and RNA pol II kinases, which promote transcription. c-Myc also promotes formation of the cap structure at the 5′ end of mRNA. The cap is 7-methylguanosine linked to the first transcribed nucleotide of RNA pol II transcripts via a 5′ to 5′ triphosphate bridge. The cap is added to the first transcribed nucleotide by the capping enzymes, RNGTT and RNMT–RAM. During the early stages of transcription, the capping enzymes are recruited to RNA pol II phosphorylated on Serine-5 of the C-terminal domain. The mRNA cap protects transcripts from degradation during transcription and recruits factors which promote RNA processing including, splicing, export and translation initiation. The proportion of transcripts with a cap structure is increased by elevating c-Myc expression, resulting in increased rates of translation. c-Myc promotes capping by promoting RNA pol II phosphorylation and by upregulating the enzyme SAHH which neutralises the inhibitory bi-product of methylation reactions, SAH. c-Myc-induced capping is required for c-Myc-dependent gene expression and cell proliferation. Targeting capping may represent a new therapeutic opportunity to inhibit c-Myc function in tumours. This article is part of a Special Issue entitled: Myc proteins in cell biology and pathology.

## Highlights

• c-Myc is a potent oncogene which drives cell proliferation by altering the gene expression landscape.
• c-Myc regulates formation of the mRNA cap, a structure critical for RNA processing and translation.
• c-Myc upregulates RNA pol II phosphorylation which recruits the “capping” enzymes.
• c-Myc upregulates SAHH which hydrolyses the inhibitory bi-products of methylation reactions.

## 1 Introduction

c-Myc is a potent cellular protein which is required for cell proliferation throughout development and in adult [1], [2]. It is upregulated in response to growth factors and transmits the signal for proliferation by regulating gene expression. c-Myc is also a prevalent oncogene which is deregulated to some extent in most human tumour types, resulting in aberrant cell proliferation [1], [2]. In recent years, many promising approaches to targeting c-Myc expression or function have been discovered, and many of these have exhibited promising results in mouse cancer models [3]. However, currently there are no therapeutic approaches in the clinic which specifically target c-Myc and therefore the need remains to understand the molecular mechanisms by which c-Myc functions. This review discusses the mechanisms by which c-Myc promotes mRNA cap formation, how this influences gene expression, and the opportunities for investigating capping as a therapeutic target to inhibit Myc function.

## 2 Discovery of c-Myc

In humans, the Myc family of proteins consists of c-Myc, N-Myc and l-Myc. c-Myc is believed to be expressed in all proliferating cells and is the Myc protein most commonly deregulated in tumours. Prior to the discovery of the mammalian Myc proteins, v-Myc was identified as one of the first-discovered viral oncogenes [4]. Subsequently, a cellular homologue of v-Myc, c-Myc, was identified as a nuclear protein. c-Myc (and all Myc proteins) were found to contain basic-helix–loop-helix leucine zipper motifs, which had been previously observed in sequence-specific DNA-binding proteins [5]. As a consequence of these observations, c-Myc was confirmed to be a transcription factor which regulates protein-encoding genes, resulting in regulation of mRNA expression in a gene-specific manner. When purified from cell extracts, c-Myc is isolated as a heterodimer with a basic-helix–loop-helix leucine zipper protein, Max. Max is required for c-Myc to bind to DNA and regulates transcription [6], [7]. The N-terminus of c-Myc binds to co-factors, including histone acetyltransferases and RNA pol II kinases, which mediate transcriptional activation and repression [2], [8]. The complex details of how c-Myc regulates transcription are tackled elsewhere in this special issue.

## 3 c-Myc is a transcriptional regulator

Our understanding of c-Myc as a transcription factor has evolved with and contributed to our understanding of mammalian transcriptional mechanisms. Initially c-Myc was identified as a transcription factor that increased and decreased expression of certain protein encoding genes. The advent of micro arrays allowed “whole genome” analysis for the first time. This revealed the surprising finding that, regardless of cell lineage, c-Myc regulates transcription of approximately 10% genome, and represses and activates genes by equivalent measure [1], [8]. c-Myc was also observed to be a relatively weak transcriptional regulator, typically activating and repressing genes by 1.5–2-fold. In the 1990s, the first evidence came that c-Myc regulates transcription elongation [9], [10]. Subsequently, most mammalian genes were found to have a pool of RNA pol II paused downstream of the promoter. Release of paused RNA pol II into elongation phase was recognised to be a rate-limiting step in transcription [11], [12]. The mechanism of RNA polymerase II pausing and release is complex and major discoveries continue to be made concerning the mechanism and its regulation. Recognition that c-Myc has a pleiotropic effect on gene expression began with the discoveries that c-Myc globally upregulates chromatin acetylation and methylation associated with transcription, and that c-Myc globally increases RNA pol II C-terminal domain Serine-2 and Serine-5 phosphorylation, events associated with transcription initiation and elongation, respectively [13], [14]. Subsequently, advanced RNA sequencing technologies have revealed that c-Myc globally amplifies transcription of the majority of RNA pol II genes [15], [16]. In addition to increasing mRNA transcription, c-Myc has been found to influence mRNA translation by promoting RNA polymerase I and III-dependent transcription, thus upregulating rRNA (ribosomal RNA) and tRNA (transfer RNA) production [17], [18], [19], [20].

## 4 mRNA 7-methylguanosine cap

**Fig. 1:**
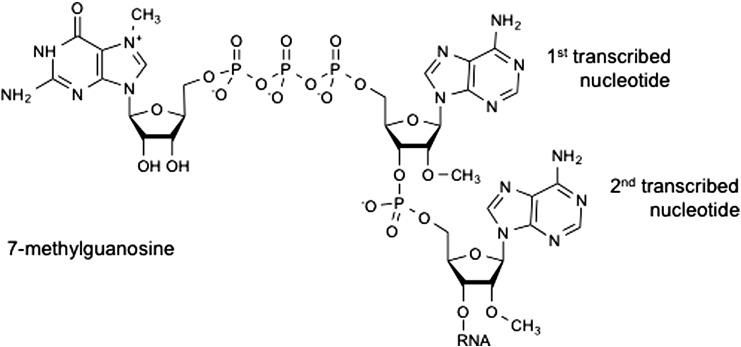
Diagram of the mRNA cap. The mRNA cap is 7-methylguanosine linked via a 5′ to 5′ triphosphate bridge to the first transcribed nucleotide. The first and second transcribed nucleotides can also be methylated on the ribose 2-hydroxyl position creating Cap1 and Cap2, respectively.

A key process during transcription of protein-encoding RNA pol II transcripts, whether c-Myc-regulated or not, is the addition of the “cap” structure to the initiating nucleotide [21], [22] (Fig. 1). The cap consists of 7-methylguanosine linked to the first transcribed nucleotide by a 5′ to 5′ triphosphate bridge (abbreviated to m7G). The cap structure is thought to be unique to the 5′ end of RNA pol II transcripts, selecting them for specific handling and processing required for their ultimate expression [23], [24], [25]. In mammals, the first and second transcribed nucleotides can also be O-2 methylated, forming part of the recognised cap structure [26].

**Fig. 2:**
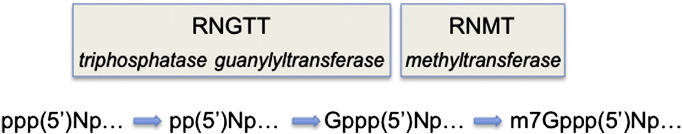
mRNA cap synthesis. Nascent RNA is transcribed with a 5′ triphosphate, denoted ppp(5′)Np, where N is the first transcribed nucleotide. RNGTT has triphosphatase and guanylyltransferase activities and catalyses addition of the inverted guanosine cap to create, G(5′)ppp(5′)Np. RNMT is the cap methyltransferase which methylates the guanosine cap on the N-7 position to create Cap 0 m7G(5′)ppp(5′)Np.

Three enzymic activities catalyse m7G formation, a triphosphatase, guanylyltransferase and methyltransferase [26] (Fig. 2). RNA is synthesised with a 5′ triphosphate, denoted ppp(5′)Np (where N is the first transcribed nucleotide). A triphosphatase removes the terminal phosphate and a guanylyltransferase catalyses addition of an inverted guanosine cap to create the cap intermediate, G(5′)ppp(5′)G. A methyltransferase methylates the inverted guanosine cap on the N-7 position to create “Cap 0”, denoted 7mG(5′)ppp(5′)Np. Methylation of the first and second transcribed nucleotides on the O-2 position of the ribose creates the structures known as Cap1 and Cap2, respectively.

Although the basic enzymic activities utilised to promote capping are similar in all eukaryotes, the genomic arrangement of the capping enzymes varies significantly in different species [26]. Since this review is concerned with the function of c-Myc in humans, we will focus on the discussion of the mammalian capping enzymes. In mammals, the triphosphatase and guanylyltransferase are contained on a single polypeptide, RNGTT (RNA guanylyltransferase and 5′ phosphatase) [27], [28], [29], and the methyltransferase is contained in a separate enzyme, RNMT (RNA guanine-7 methyltransferase) [29], [30] (Fig. 2). RNMT is isolated from mammalian cells in a complex with an activating subunit, RAM (RNMT-activating mini-protein) [31], [32]. The guanylyltransferase reaction is reversible whereas N-7 methylation is not, thus N-7 methylation “locks-in” the cap structure. CMTR1 and CMTR2 methylate the first and second transcribed nucleotides, although not all transcripts receive this modification [33], [34]. Furthermore, the precise mechanisms of function of CMTR1 and CMTR2 are in the early days of characterisation and not mentioned further here.

The cap methylation reaction is not readily reversible, however the entire cap can be removed by a variety of “decapping enzymes” [35], [36], [37]. mRNA is unstable without the cap and therefore decapping either initiates degradation or is a later stage of the degradation process. The decapping enzyme complexes can potentially act on all transcripts but in vivo can exhibit specificity for certain transcripts. Furthermore, various auxiliary proteins can increase decapping enzyme activity and direct the decapping complexes towards specific transcripts, thus facilitating cellular regulation of the process.

## 5 Capping and transcription

Not only does formation of the cap occur during transcription, it is integral to the process. RNA is vulnerable to degradation during the early stages of transcription and addition of the cap structure protects RNA pol II transcripts from attack by exonucleases. Addition of the cap thus passively permits transcripts to be synthesised [38], [39].

Capping occurs shortly after transcription initiation and is restricted to RNA pol II transcripts since the capping enzymes RNGTT and RNMT–RAM are only recruited to this polymerase [40], [41]. The RNA pol II large subunit C-terminal domain (CTD) is a recruitment platform for enzymes and factors which regulate transcription and modify RNA, including the capping enzymes [12], [42]. Recruitment of factors to the CTD is co-ordinated by a series of phosphorylation events and other post-translational modifications. RNGTT and RNMT–RAM are recruited at the initial phases of transcription when the RNA polymerase II CTD is phosphorylated on Serine-5 [43], [44]. RNGTT is activated by interaction with Serine-5 phosphorylated CTD [45], [46], and by interaction with the transcription elongation factor hSPT5 [47].

## 6 mRNA cap function

The mechanism of capping can have a “pro-active” effect on transcription. In yeast species, all three capping enzymes have been demonstrated to influence transcription, some independent of enzymic activity [40], [41], [48]. Recruitment of the capping enzymes has been demonstrated to be integral to the switch from transcription initiation to elongation [48], [49]. In mammals, only RNGTT has been demonstrated to promote transcription to date [50]. In vitro, RNGTT was demonstrated to relieve repression by NELF (negative elongation factor), indicating a role for the capping enzyme in release of RNA pol II from pausing/promoter clearance. It is not a forgone conclusion that RNMT–RAM will be found to influence transcription in mammals. There are key differences between yeast and mammals in the processes of transcription initiation and elongation; release of polymerase from pausing is more complex and heavily regulated in mammals. Furthermore, the capping enzymes are configured differently in yeast and mammals [26]. In yeast species, there are three distinct capping enzymes, whereas in mammals the triphosphatase and guanylyltransferase are found on the same peptide, RNGTT. The cap methyltransferase, RNMT, has an activating subunit, RAM, in mammals which is not found in yeast [31]. In addition the mammalian cap methyltransferase has an N-terminal domain which is required for efficient recruitment to RNA pol II which is not present in yeast species [44].

As described above, the cap is added to the transcripts shortly after initiation and remains throughout its lifetime. The cap directs RNA pol II transcripts to be processed quite distinctly from RNA pol I and III transcripts. The cap structure mediates processing events ultimately required for translation, including, splicing, mRNA export, polyadenylation and translation initiation [23], [24], [25]. Factors including CBC (cap binding complex) and eIF4E (Eukaryotic initiation factor 4E) bind to the cap structure and recruit the proteins required for processing and translation initiation. Many studies have found the cap or cap-binding protein complexes to have gene-specific effects on RNA processing and translation [23], [24], [25]. Modern sequencing technologies are beginning to provide the experimental means of determining the precise function of the cap genome-wide. For example, which transcripts (and which exons) require methylation of the cap for splicing and which transcripts require methylation of the cap for export? Since most gene expression processes are tightly coupled, discerning direct from indirect effects of the cap in endogenous gene expression is experimentally challenging. This problem is compounded in mammalian systems by protein knock-down/knock-out technologies requiring many hours or days to become effective. Precise determination of the direct versus indirect effects of the cap on gene expression is likely to be improved by the development of specific inhibitors of the capping enzymes, RNGTT and RNMT–RAM.

## 7 c-Myc regulates capping

**Fig. 3:**
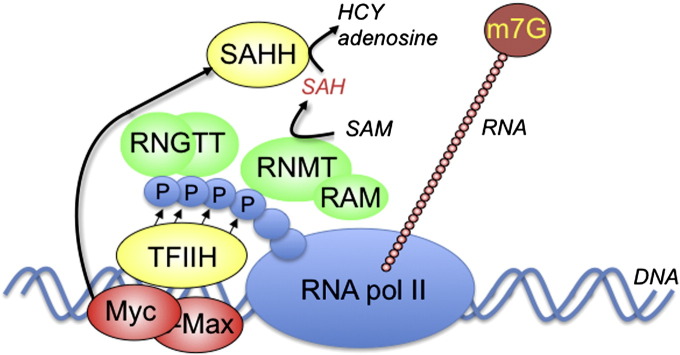
c-Myc regulated mRNA cap synthesis. c-Myc-Max binds proximal to transcription initiation sites, promotes TFIIH recruitment and RNA pol II phosphorylation. RNGTT and RNMT–RAM are recruited to the phosphorylated C-terminal domain of RNA pol II, where they catalyse mRNA cap (m7G) synthesis. The methylation reaction uses the methyl donor, SAM (s-adenosyl methionine), and produces the inhibitory bi-product SAH (s-adenosyl homocysteine). c-Myc upregulates SAHH (S-adenosyl homocysteine hydrolase) which catalyses hydrolysis of SAH producing adenosine and homocysteine (HCY).

In 2007, c-Myc was found to promote formation of the cap structure, thus synergistically promoting transcription and translation of RNA pol II transcripts (Fig. 3) [14], [51], [52]. This was an unexpected finding since capping was widely regarded as a “house-keeping” event, evolved to select RNA pol II transcripts for specific processing and handling. As described earlier, capping and transcription are mechanistically coupled processes and c-Myc promotes these processes by partially overlapping mechanisms [14], [51], [52]. c-Myc binds proximal to promoters, most frequently to conserved binding sites downstream of transcription initiation sites [53], [54], [55]. c-Myc promotes TFIIH recruitment to DNA by direct interaction with the kinase complex, and probably also by relaxing the chromatin structure [13], [14], [56]. RNA pol II phosphorylation promotes recruitment of the capping enzymes, RNGTT and RNMT, and activation of RNGTT [12], [42]. For the transcripts investigated, this correlates with increased capping, translation and protein expression [14].

The mechanism of c-Myc-dependent cap formation is also dependent on upregulation of the enzyme, SAHH (S-adenosyl homocysteine hydrolase) [52]. Methylation reactions utilise the methyl donor, S-adenosyl methionine (SAM) and produce the bi-product S-adenosyl homocysteine (SAH) [57]. SAH inhibits methylation reactions by competing with SAM for the active site. c-Myc-dependent cap formation was found to be dependent on upregulation of SAHH which hydrolyses SAH to the neutral products homocysteine and adenosine. c-Myc-dependent cap methylation is particularly sensitive to SAHH levels; inhibition of c-Myc-dependent SAHH upregulation to basal levels reduces c-Myc dependent gene expression, cell proliferation and cell transformation [52]. Many methyltransferase have been found to be dependent on SAHH levels, including the Adenosine N-6 methyltransferase [58]. Since c-Myc significantly increases SAHH expression it seems likely that c-Myc will influence other RNA methylation events.

Which step in cap formation does c-Myc regulate; addition of the guanosine cap catalysed by RNGTT, N-7 methylation of the guanosine cap catalysed by RNMT, or both? Since c-Myc increases RNA pol II phosphorylation, it could potentially increase recruitment of RNGTT and RNMT, i.e. promote guanosine cap addition *and* methylation of the guanosine cap. We know that c-Myc increases the proportion of transcripts with a 7-methylguanosine cap (m7G(5′)ppp(5′)Np), but currently we do not know whether the transcripts without this structure have the inverted guanosine cap added (G(5′)ppp(5′)N), or not (ppp(5′)N). Although uncapped transcripts are unstable at the initial stages of transcription, during transcription elongation they rapidly become coated in proteins and gain secondary structure which probably stabilises the transcript. The question of whether unmethylated transcripts have a guanosine cap or not is important since some of the cap-binding proteins have affinity for the guanosine cap, albeit reduced [23], [24], [25]. Therefore transcripts that have a guanosine cap are probably more translation-competent than those without. In yeast, incompletely capped transcripts have also been observed, particularly under conditions of nutritional stress [59], [60]. Furthermore, incompletely capped transcripts have also recently been observed in other mammalian systems [61].

We do not understand why certain transcripts are more receptive for c-Myc-dependent capping than others. This is likely to be governed by features of the gene or transcript. For example, certain chromatin configurations may result in more access of RNA pol II to the capping enzymes, or faster transcription rates may reduce the time the capping enzymes have access to the transcript. Alternatively, 5′ untranslated regions of transcripts which hold significant secondary structure may have less access to the capping enzymes. Whole genome analysis of the recruitment of the capping enzymes may begin to distinguish between these possibilities.

## 8 Do other transcription factors regulate capping?

Is c-Myc a special case in being a transcription factor which promotes mRNA cap methylation? Probably not. The Myc proteins, c-Myc and N-Myc both up regulate formation of the cap [14], [51], [52]. E2F1 is another transcription factor which promotes formation of the cap in a mechanism dependent on RNA pol II phosphorylation [51], [62]. It seems likely that any transcription factor which influences RNA pol II phosphorylation will promote capping of a subset of transcripts. Furthermore, since mRNA cap methylation is highly dependent on SAHH, any signalling pathways that regulate SAHH expression or activity are likely to influence mRNA cap methylation.

## 9 Targeting capping

Mechanisms which c-Myc utilises to promote cell proliferation have the potential to be targeted therapeutically to inhibit deregulated cell proliferation in tumour cells [3]. The question of whether mRNA cap synthesis should be investigated as a potential therapeutic target can be addressed from both biological and practical perspectives.

From a biological perspective, the current published research suggests that capping should be considered as a therapeutic target. Inhibition of SAHH specifically inhibits the proliferation of cells harbouring deregulated c-Myc [52]. Furthermore, upregulating expression of RNMT is sufficient to transform mammary epithelial cells [63]. Although abolishing capping is likely to be lethal in all mammalian cell lineages, mild inhibition of capping may selectively target the most transcriptionally active cancer cells. Most current cancer therapeutics target major cellular pathways, which if abolished would result in cell lethality, but when attenuated have selectivity for transformed cells.

From a practical perspective, mRNA cap synthesis is catalysed by enzymes, which have the potential to make good targets since they already bind to small molecules (ligands) in their active sites. RNMT would appear to be a better potential target than RNGTT since the latter has a highly charged active site, and is therefore likely to bind to charged/polar molecules, which are resistant to passage across the plasma membrane. Compounds that inhibit the active site of RNMT are currently available, but lack specificity [52]. Although the vast majority of inhibitors target enzyme active sites, protein–protein interactions are being increasingly utilised as therapeutic targets. As described earlier, RNMT exists in a heterodimer with an activating subunit RAM (RNMT-activating miniprotein) [31], [32]. RAM activates RNMT six-fold in vitro and disrupting the interaction between RNMT and RAM may provide the possibility of reducing rather than annihilating the cellular cap methyltransferase activity, thus limiting toxicity.

## 10 Conclusion and remarks

Much remains to be discovered about the mechanism and biological significance of c-Myc-regulation of mRNA cap synthesis. c-Myc is one of the first examples of a cellular signalling protein regulating mRNA cap synthesis. Given the overlapping mechanisms of transcription and capping, it seems likely that many transcriptional regulators will promote capping to some extent. It is also possible that other signalling pathways will be found to regulate capping by regulating capping enzyme expression or activity.
